# Invasion of moso bamboo into a Japanese cedar plantation affects the chemical composition and humification of soil organic matter

**DOI:** 10.1038/srep32211

**Published:** 2016-08-25

**Authors:** Hsueh-Ching Wang, Guanglong Tian, Chih-Yu Chiu

**Affiliations:** 1 Biodiversity Research Center, Academia Sinica, Nankang, Taipei 11529, Taiwan; 2 Environmental Monitoring and Research Division, Monitoring and Research Department, Metropolitan Water Reclamation District of Greater Chicago (MWRD), Lue-Hing R&D Laboratory, 6001 W. Pershing Road, Cicero, IL 60804, USA

## Abstract

Bamboo, which has dense culms and root rhizome systems, can alter soil properties when it invades adjacent forests. Therefore, this study investigated whether bamboo invasions can cause changes in soil organic matter (SOM) composition and soil humification. We combined solid-state ^13^C NMR spectroscopy and chemical analysis to examine the SOM in a Japanese cedar (*Cryptomeria japonica*) and adjacent bamboo (*Phyllostachys edulis*) plantation. Bamboo reduced soil organic C (SOC) content, compared to the cedar plantation. The value of ∆logK (ratio of absorbance of humic acids at 400 and 600 nm) was cedar > transition zone > bamboo soils. Our results indicated that bamboo increased SOM humification, which could be due to the fast decomposition of bamboo litter with the high labile C. Furthermore, intensive management in the bamboo plantation could enhance the humification as well. Overall, litter type can control an ecosystem’s SOC nature, as reflected by the finding that higher labile C in bamboo litter contributed the higher ratios of labile C to SOC and lower ratios of recalcitrant C to SOC in bamboo soils compared with cedar soils. The invasion of bamboo into the Japanese cedar plantation accelerated the degradation of SOM.

Soil organic matter (SOM) is the largest carbon (C) pool in terrestrial ecosystems. The stability and decomposability of SOM plays an important role in regulating regional or local C cycles, and eventually the CO_2_ exchange between the atmosphere and soils^1^. The SOM stability and decomposability are associated with the local vegetation type, climate, soil’s nutrient status, and land use strategy^1^,^2^. Therefore, changes in vegetation type can alter litter quality, which affects SOM accumulation, and its composition and structure^2^,^3^. Furthermore, vegetation changes can also alter soil microbial community structure and activity^4^, which can indirectly affect the quantity and composition of SOM in an ecosystem. Consequently, these alterations in SOM and microbial composition due to vegetation changes can affect soil respiration rates and the CO_2_ emission from the soil^5^.

Humification is a soil process that transforms and converts organic matter to humic substances, which represent a pool of strongly altered soil organic matter^6^. Environmental characteristics such as climate and vegetation type influence the humification process, as well as the composition and structure of soil humic substances^6^,^7^. Understanding the effect of vegetation change on SOM humification and its chemical composition is important for evaluating the ecological impacts of such changes on soil C pool, especially within the context of terrestrial C fluxes.

Bamboo is one of the most important commercially planted species in East Asia^8^. Because of its unique root rhizome system, from which shoots and culms emerge, bamboo is one of the fastest-growing plant species in the world^9^. This extensive rhizome system also makes bamboo spread laterally, enabling it to invade into surrounding forests or agricultural systems^10^. Bamboo expansion can subsequently result in the decline of plant species diversity and affect plant community function^8^,^11^. For example, bamboo invasions to tree stands can cause high mortality rates of tree seedlings^12^, or alter plant communities by replacing abandoned stands^10^,^11^. Furthermore, the invasive rhizome system of bamboo can change soil chemical properties, such as soil pH and exchangeable cation content^13^. Changes in litter quality and soil characteristics due to bamboo invasion can consequently lead to the increase of easily decomposable organic matter, a reduction of stress for microbes^4^, and an increase in soil bacterial diversity^4^,^14^. However, little is known about how bamboo invasion in sub-alpine ecosystems influence SOM quantity and chemical composition.

Solid-state^13^C CP-MAS nuclear magnetic resonance (NMR) spectroscopy is widely used as a non-destructive method to investigate the chemical composition of SOM^15^,^16^. The chemical shifts of C functional groups of SOM resulting from these analyses represent different components of recalcitrant and labile C^15^,^17^. As such, NMR chemical composition of SOM has been used to evaluate litter decomposition and degree of humification^3^,^17^. We hypothesized that bamboo invasions into cedar plantations can cause an increase in the degree of SOM humification, due to the faster decomposition of bamboo litter. The objective of this study was to investigate the change in SOM humification and composition after bamboo invaded a Japanese cedar plantation, using both chemical analyses and solid-state ^13^C NMR spectroscopy with cross-polarization and magic-angle spinning (CP-MAS ^13^C NMR).

## Results

### Soil properties

The soil texture in the studied Japanese cedar plantation was classified as clay, whereas the soil texture in the transition zone and bamboo plantation was classified as clay loam (Table 1). The pH significantly differed among the three vegetation types, being highest in the transition zone and lowest in the Japanese cedar plantation. The SOC was highest in the Japanese cedar plantation and lowest in the bamboo plantation. Furthermore, total N content was highest in the soil of the Japanese cedar plantation, and lowest in the transition zone. The soil C/N ratio was also highest in the Japanese cedar plantation, and lowest in the transition zone. Humic acids had a lower ∆logK in bamboo than Japanese cedar soils.

### The pattern of ^13^C NMR C functional groups

**Figure 1:**
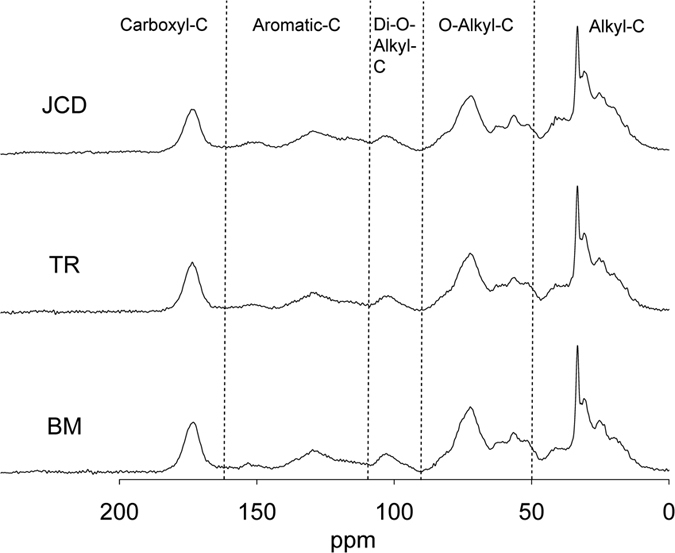
The solid state CP-MAS ^13^C NMR spectra of humid acids in surface soil in the Japanese cedar plantation (JCD), the transition zone (TR), and the bamboo plantation (BM). The chemical shift regions are: 0–50 ppm (alkyl-C), 50–90 ppm (O-alkyl-C), 90–110 ppm (Di-O-alkyl-C), 110–165 ppm (aromatic-C) and 165–190 ppm (Carboxyl-C).

The main resonances of surface soil occurred at 30, 71, 104, 127, and 172 ppm for five C functional groups, respectively (Fig. 1). Among the three types of plantations, Alkyl-C abundance was significantly higher in the litter of the Japanese cedar than in the bamboo plantation, but not in the surface soil (Table 2). The O-alkyl-C contents were lowest in the Japanese cedar planation’s litter and soil, and highest in the bamboo plantation. Di-O-alkyl-C content was similarly lowest in the Japanese cedar plantation litter, and highest in the bamboo in litter. Furthermore, aromatic-C content was highest in the transition zone litter, and lowest in the bamboo plantation litter, whereas for the soil it was highest in the Japanese cedar plantation and lowest in the bamboo plantation. Carboxyl-C content was lowest in the soil of the Japanese cedar plantation and highest in the transition zone, but the litter values were not significantly different among the vegetation types. The ratio of Alkyl-C to O-alkyl-C and Di-O-alkyl-C together (A/O-A) was highest in the Japanese cedar litter, and lowest in the bamboo litter. The soil A/O-A ratio did not differ among the three vegetation types. The aromaticity in the litter was highest in the transition and lowest in bamboo litter, whereas for the soils it was highest in the Japanese cedar plantation and lowest under the bamboo.

### The labile and recalcitrant pools of SOC

The contents of soil labile C (LPI-C and LPII-C) and recalcitrant C (RP-C) were highest in the Japanese cedar plantation and lowest in the bamboo plantation (Table 3). Considering that SOC was different among the three vegetation types, we examined the ratios of various C fractions to soil total organic C content. The ratio of LPI-C and LPII-C to soil total organic C (LPI-C/TC and LPII-C/TC) were lowest in the Japanese cedar plantation, and highest in the bamboo plantation, although the ratio of RP-C to soil total organic C (RP-C/TC) was highest in the Japanese cedar plantation, and lowest in the bamboo plantation.

## Discussion

### The effect of bamboo invasion on soil properties

The clay content was higher in Japanese cedar than bamboo plantation (Table 1). This difference might be associated with enhanced soil erosion and clay loss due to the disturbance of frequent bamboo shoot harvesting. By forming an association with SOM, clay can physically protect soil organic matter (SOM), thus slowing SOM turnover^18^. Therefore, the loss of clay in the bamboo plantation possibly promoted SOM decomposition there. In addition, the herbaceous bamboo litter is easily decomposable, causing less organic matter to remain in the soil, which also can explain the lower SOC and TN in the bamboo soils compared with the Japanese cedar plantation. Furthermore, soil pH was high in the bamboo plantation, and bamboo invasion can increase the base saturation of the surface soil, resulting in an increase in soil pH^13^. The increase in soil pH could furthermore be a consequence of the decrease in SOM after the bamboo invasion. Overall, the bamboo invasion had an effect on a range of soil properties.

### Chemical composition of SOM and humification

The O-alkyl-C and Di-O-alkyl-C dominant in hydrolysable polysaccharides (cellulose and hemicellulose) were the highest in bamboo litter (Table 2), indicating that bamboo litter contained more easily decomposable substances. The Japanese cedar litter contained substances that are more difficult to decompose, as it had a high proportion of alkyl-C and aromatic-C, which were dominant in more recalcitrant substances, such as lignin, tannin, surface waxes, lipids, cutins, and resins.

The value of ∆logK generally decreases when SOM humification increases, and it is used as an index of humification^19^. The lower value of ∆logK in the bamboo soils indicated that the degree of SOM humification increased from Japanese cedar to bamboo plantation (Table 1). The A/O-A ratio and aromaticity, which increase with the process of humification, are regarded as another index of the degree of humification^17^,^20^. Therefore, from the NMR C functional groups, SOM in the bamboo soil had the lowest level of humification, as it had the lowest A/O-A ratio and aromaticity. This contradictory trend can be explained by the lower A/O-A ratio and aromaticity in the bamboo litter (Table 2). In general, litter and root production is the major sources of SOM, and the high ratio of litter to root production (1.07-3.57) indicated litter had a substantial contribution to SOM pool^21^,^22^,^23^. Therefore, it seems that the large differences in litter quality between bamboo and Japanese cedar can outweigh effects of SOM structure and property.

This litter control of SOM by bamboo and cedar vegetation was reflected in the observed changes in labile and recalcitrant C pools. The ratios of labile C to soil total organic C (LPI-C/TC and LPII-C/TC) in bamboo soils were higher than those in Japanese cedar soil (Table 3), which could be explained by the high labile organic substances in the herbaceous bamboo litter. Likewise, the high ratio of recalcitrant C to soil total organic C (RP-C/TC) in Japanese cedar plantations soils is most probably associated with the high content of recalcitrant substances (alkyl-C and aromatic-C) in the litter at that site. A different study similarly found that high A/O-A ratio in conifer litter could lead to higher A/O-A ratio in conifer soils, compared to broadleaf soils^3^. Another similar study also showed that vegetation types, through controlling the litter physicochemical quality and local microbial communities, dominated the process of decomposition and behaviour of C functional groups^24^.

In addition to the litter of bamboo, different human management can also have an influence on SOM humification. Compared to the negligible disturbance in Japanese cedar plantation, farmers harvested bamboo shoots frequently. The intensive bamboo shoot harvesting can stimulate the decomposition of bamboo litter, and accelerate the SOM humification in the bamboo plantation. Li *et al.*^25^ found that long-term tillage could accelerate the decomposition rate of SOM, and decrease both labile and recalcitrant organic C pools in a moso bamboo plantation. The intensive management in the bamboo plantation could have stimulated the decomposition of carbohydrates and increase the aromaticity. Furthermore, SOM humification could have been accelerated with cultivation duration^25^,^26^. In addition, intensive bamboo management could stimulate the mineralization of labile organic C, and increase soil CO_2_ emissions^27^,^28^. Overall, the composition of litter from three types of vegetation was critical to influence the humification, which might be underestimated in bamboo soil. The increase in SOM humification by intensive cultivation could accompany accelerated SOM degradation and increase the soil CO_2_ emission from the bamboo plantation.

Bamboo plantation and intensive management could decrease SOC and total N contents in sub-alpine ecosystems, probably resulting in enhanced CO_2_ emissions. The lower value of ∆logK in the bamboo plantation indicated a high degree of SOM humification in the bamboo soil, although its A/O-A ratio and aromaticity indicated the opposite. Litter under the Japanese cedars had more recalcitrant substances (as showed in high A/O-A ratio and aromaticity) than bamboo had, which made the Japanese cedar soil contain a high A/O-A ratio and aromaticity. The highest LPI-C/TC and LPII-C/TC, but the lowest RP-C/TC in the bamboo plantation soil reflected the high labile C in bamboo litter. The high SOM humification in the bamboo plantation was additionally a consequence of the intensive bamboo shoot harvesting, which enhanced the decomposition of SOM. Overall, our findings showed that the humification of bamboo soils might be underestimated if litter C composition is not considered. Bamboo invasion could enhance SOM humification and accelerate soil degradation by changing litter composition, which would influence soil microbial activity and SOC accumulation.

## Methods

### Study site

This study was carried out at Shanlinshi in central Taiwan (120°46′06″ E, 23°40′42″N), which has a humid subtropical climate. The mean annual temperature here is about 17 °C, and the total annual precipitation is about 2600 mm. Furthermore, the elevation of the study site is 1350 m. The study site is owned by the Experimental Forest from the College of Bio-Resources and Agriculture, National Taiwan University (NTU), located in Nantou County, central Taiwan. Japanese cedars (*Cryptomeria japonica*) were planted here about 40 years ago, after the natural camphor forest was logged. Furthermore, a plantation of moso bamboo (*Phyllostachys edulis*), a temperate species of giant timber bamboo, was established adjacent to the Japanese cedar plantation at approximately the same time. The transition zone between the Japanese cedar and the moso bamboo spans about 30 to 50 m in width, and contains a mixture of both species. The transition zone was established by bamboo culms that invaded the cedar plantation. Here, the dense culms and rhizome system of the bamboo restrict the understory growth, similar to the bamboo plantation, and the fast-growing bamboo almost reaches the same height as the top of the cedar plantation canopy. Overall, the bamboo culm density decreased from the boundary of the bamboo plantation to the inner part of the cedar plantation. Both the moso bamboo plantation and the transition zones were considered to be bamboo-invaded plots. Furthermore, the soil in the moso bamboo plantation is more disturbed due to the frequent activity there, with farmers cutting the bamboo stems and digging in the soil to harvest bamboo shoots. In contrast, the soil in Japanese cedar plantation is less disturbed, and has a relatively dense understory. A more detailed description of the study site can be found elsewhere^14^.

### Soil sampling and analysis

Six replicated transect lines from the bamboo to the cedar plantation were set up with intervals larger than 50 m. Three sampling plots of 25 m × 25 m in three vegetation types (cedar, bamboo, and transition) were established per transect line. In February 2011, the surface soil (0–10 cm depth) was sampled with a soil auger (diameter of 8 cm and length of 10 cm). Five soil cores were combined as a composite sample per plot. The soil samples were cleared from visible roots and litter residues, and subsequently air-dried and ground through a 2-mm sieve. The pH was measured at a 1:1 soil-to-water ratio. Soil texture was measured as particle size distribution by the pipette method. Soil organic C and total N were measured by the combustion method using an NCS elemental analyser (Model NA1500 Fisons, Italy). Forest floor litter was collected in a plot of 0.5 m × 0.5 m per transect, and litter was oven-dried and ground as powder for NMR spectroscopy analysis.

### Extraction of soil humic acids for NMR and photometric analysis

The humic substances were extracted from the samples with 0.1 M NaOH in a soil-to-extractant ratio of 1:10, under an N_2_ atmosphere. The alkaline supernatant was centrifuged at 15,000 × *g* for 20 min, and was subsequently acidified to pH 1.0 with 6 M HCl and left at room temperature for 12–16 h to precipitate. After this, the coagulated materials were removed from the supernatant by centrifugation at 5,500 × *g* for 15 min. The humic acids were first purified with a 0.1 M KOH and 0.3 M KCl mixture, and subsequently centrifuged at 10,000 × *g* for 10 min. The supernatant was then acidified to pH 1.0 with 6N HCl, and centrifuged again at 5,500 × *g* for 15 min. A 0.1 M HCl-0.3 M HF mixture was used to further purify the humic acids, and this mixture was centrifuged at 5,500 × *g* for 15 min. The humic acids were collected after the fractions were condensed, and freeze-dried for ^13^C CP-MAS NMR spectroscopy.

Another protocol was prepared for the extraction of humic acids for photometric analysis. The air-dried soil samples (1 g) were mixed with 30 ml 0.1N NaOH and shaken for 30 min at 100 °C. Subsequently, 2 ml Na_2_SO_4_ was added, and the samples were centrifuged at 10,000 × *g* for 15 min. The precipitates were flushed with 20 ml 0.1N NaOH, and the humic acid fraction was obtained by acidifying 100 ml of extractant with 1 ml concentrated H_2_SO_4_ (98%) and the precipitates were collected by centrifugation. The precipitates were subsequently dissolved in 30 ml 0.01N NaOH to determine the absorbance of humic acids at 400 and 600 nm using a spectrophotometer (Hitachi U-2000)^6^. The ratio of absorbance of humic acids at 400 and 600 nm, defined as ∆logK, is an inverse index of condensation of the aromatic network in humic acid macromolecules and the degree of humification^19^.

### Solid-state ^13^C NMR spectroscopy

Three samples of soil humic acids and powdered litters per vegetation type were examined to determine chemical shifts by solid-state ^13^C CP-MAS NMR spectroscopy (BRUKER DSX 400-MHz solid-state NMR, Germany) with a 7-mm-diameter sample tube. Data acquisition happened at the conditions of spectrometer frequency 100.46 MHz, spectra width 20,000 Hz, spinning speed 7,000 Hz, contact time 6 ms, pulse delay time 1 s, and with 8,200 scans^3^,^29^. The five chemical shifts were 0–50 ppm (alkyl-C), 50–90 ppm (O-alkyl-C), 90–110 ppm (Di-O-alkyl-C), 110–165 ppm (aromatic-C), and 165–190 ppm (carboxyl-C)^15^,^29^. The relative intensities of chemical-shift areas were integrated from the ^13^C CP-MAS NMR spectra over the given chemical-shift ranges to the total area of the spectrum. We calculated the ratio of alkyl-C to O-alkyl-C and Di-O-alkyl-C (A/O-A) as an index of the degree of humification^20^. The aromaticity was calculated as another index of degree of humification using the ratio of aromatic-C (110–165 ppm) to the summed areas of alkyl-C, O-alkyl-C, Di-O-alkyl-C, and aromatic-C (0–165 ppm)^30^.

### Determination of labile and recalcitrant C

The two-step acid hydrolysis with sulphuric acid (H_2_SO_4_) as the extractant was used to isolate and quantify labile and recalcitrant SOC^31^. The SOM in a 0.5 g soil sample was hydrolysed with 20 ml of 5N H_2_SO_4_ at 105 °C for 30 min in Pyrex flasks with Allihn condensers. The hydrolysated SOM in each sample was collected through centrifugation and decantation of the supernatant. The residue was flushed with 20 ml of de-ionized water, and the extract was added to the previous hydrolysate. The C in the hydrolysate was considered to be the labile C pool I (LPI-C). The remaining residual SOM was hydrolysed with 2 ml of 26N H_2_SO_4_ overnight at room temperature, whilst continuously shaking. The concentration of the sample was brought down to 2N by dilution, and the sample was subsequently hydrolysed for 3 h by heating at 105 °C. De-ionized water (26 ml) was used to flush the residues to the hydrolysate. The second hydrolysate was considered the labile C pool II (LPII-C). The remaining SOM in the sample, regarded as the recalcitrant C pool (RP-C), was washed with 30 ml de-ionized water, and dried at 60 °C in a pre-weighed crucible. The LPI-C and LPII-C were quantified using a total organic C analyser (Model 1010, O.I. Analytical, Texas), whereas the RP-C was determined with an NCS elemental analyser.

### Statistical analysis

One-way ANOVAs were used to determine significance of effect of vegetation type on soil physical and chemical properties, ^13^C NMR C functional groups, and soil labile and recalcitrant C pools. Tukey’s HSD comparisons were performed to determine which vegetation types significantly differed from each other. Significant differences were reported at 5% level. All statistical analyses were conducted with the Minitab 16 Statistical Software (Minitab Inc., PA, USA).

## Additional Information

**How to cite this article**: Wang, H.-C. *et al.* Invasion of moso bamboo into a Japanese cedar plantation affects the chemical composition and humification of soil organic matter. *Sci. Rep.* **6**, 32211; doi: 10.1038/srep32211 (2016).

## Figures and Tables

**Table 1:** Average slope, soil texture, pH, organic C (Org. C), total N (TN), C:N ratio (C/N), and ∆logK of humic acids in the surface soil (0–10 cm) per vegetation type ± standard error (n = 6).

Vegetation type		Soil texture (%)			pH (H_2_O)	Org. C (%)	TN (%)	C/N	∆logK
	Slope (%)	Sand	Silt	Clay					
Japanese cedar	20	24.7	35	40.7	3.36 ± 0.05 b	21.5 ± 1.46 a	1.33 ± 0.07 a	16.1 ± 0.39 a	0.82 ± 0.007 a
Transition zone	21	31	35	34.2	4.38 ± 0.25 a	9.36 ± 0.93 b	0.61 ± 0.10 b	10.9 ± 0.57 b	0.66 ± 0.004 b
Bamboo	10	33.7	38	28.1	4.13 ± 0.05 a	8.00 ± 0.40 b	0.67 ± 0.03 b	12.0 ± 0.33 b	0.61 ± 0.003 c
*p*-value					0.001	<0.001	<0.001	<0.001	<0.001

The different letters indicate significant differences per soil variable between the vegetation types according to Tukey’s HSD comparisons at *p* = 0.05, and the bottom row represents the p-value of the one-way ANOVA results.

**Table 2:** The proportions of NMR C functional groups of humic acids in surface soil (0–10cm) per vegetation type ± standard error (n = 3).

Vegetation	Carbon functional groups (%)					A/O-A ratio	aromaticity
	Alkyl-C	O-alkyl-C	Di-O-alkyl-C	Aromatic-C	Carboxyl-C		
Litter							
Japanese cedar	24.0 ± 0.34 a	45.3 ± 0.71 b	9.85 ± 0.32 b	16.1 ± 0.56 a	4.82 ± 0.16	0.43 ± 0.01 a	16.9 ± 0.61 a
Transition	22.4 ± 0.59 a	46.3 ± 0.76 b	9.88 ± 0.25 b	16.5 ± 0.50 a	4.99 ± 0.10	0.40 ± 0.02 a	17.3 ± 0.51 a
Bamboo	13.2 ± 0.33 b	56.5 ± 0.53 a	12.9 ± 0.17 a	12.6 ± 0.52 b	4.83 ± 0.19	0.19 ± 0.00 b	13.2 ± 0.57 b
*p*-value	<0.001	<0.001	<0.001	0.004	0.690	<0.001	0.004
Soil							
Japanese cedar	36.6 ± 0.70	29.7 ± 0.73 b	6.01 ± 0.22	18.2 ± 0.35 a	9.51 ± 0.01 b	1.03 ± 0.05	20.1 ± 0.39 a
Transition	35.8 ± 1.12	31.1 ± 0.55 ab	5.84 ± 0.15	16.2 ± 0.33 b	11.0 ± 0.29 a	0.97 ± 0.05	18.3 ± 0.43 b
Bamboo	33.8 ± 0.41	33.3 0.04 a	5.84 ± 0.03	16.2 ± 0.38 b	10.9 ± 0.18 a	0.86 ± 0.01	18.1 ± 0.42 b
*p*-value	0.105	0.008	0.679	0.011	0.003	0.063	0.026

The different letters indicate significant differences per variable between the vegetation types according to Tukey’s HSD comparisons at *p* = 0.05, and the bottom row represents the *p*-value of the one-way ANOVA results.

**Table 3:** The labile C pool I (LPI-C) and II (LPII-C), the recalcitrant C pool (RP-C), and the ratios of these SOC pools to total C per vegetation type ± standard error (n = 6).

Elevation	LPI-C (g C kg^−1^)	LPII-C (g C kg^−1^)	RP-C (g C kg^−1^)	LPI-C/TC	LPII-C/TC	RP-C/TC
Japanese cedar	58.9 ± 3.3 a	20.5 ± 2.2 a	187.8 ± 8.3 a	0.277 ± 0.014 b	0.095 ± 0.006 b	0.88 ± 0.026 a
Transition	41.5 ± 3.0 b	10.9 ± 1.2 b	75.5 ± 12.8 b	0.452 ± 0.025 a	0.117 ± 0.006 a	0.781 ± 0.060 ab
Bamboo	38.3 ± 1.7 b	10.2 ± 0.8 b	54.6 ± 3.7 b	0.480 ± 0.010 a	0.127 ± 0.005 a	0.681 ± 0.016 b
*p*-value	<0.001	<0.001	<0.001	<0.001	0.005	0.007

The different letters indicate significant differences per soil variable between the vegetation types according to Tukey’s HSD comparisons at *p* = 0.05, and the bottom row represents the p-value of the one-way ANOVA results.
